# Experimental contextuality in classical light

**DOI:** 10.1038/srep44467

**Published:** 2017-03-14

**Authors:** Tao Li, Qiang Zeng, Xinbing Song, Xiangdong Zhang

**Affiliations:** 1 Beijing Key Laboratory of Nanophotonics & Ultrafine Optoelectronic Systems, School of Physics, Beijing Institute of Technology, 100081, Beijing, China; 2 Beijing Key Laboratory of Nanophotonics & Ultrafine Optoelectronic Systems, Kunming Institute of Physics, Kunming, 650223, China

## Abstract

The Klyachko, Can, Binicioglu, and Shumovsky (KCBS) inequality is an important contextuality inequality in three-level system, which has been demonstrated experimentally by using quantum states. Using the path and polarization degrees of freedom of classical optics fields, we have constructed the classical trit (cetrit), tested the KCBS inequality and its geometrical form (Wright’s inequality) in this work. The projection measurement has been implemented, the clear violations of the KCBS inequality and its geometrical form have been observed. This means that the contextuality inequality, which is commonly used in test of the conflict between quantum theory and noncontextual realism, may be used as a quantitative tool in classical optical coherence to describe correlation characteristics of the classical fields.

Contextuality is a curious property in quantum world. In classical world, an observable is predefined and has a joint probability distribution when it is measured with the other observables. This observable is independent of other compatible observables that are measured simultaneously with it. This independency is called noncontextuality. Kochen and Specker proposed the original contextual theory in 1967^1^. The theory gives the conflict between quantum mechanics and noncontextuality reality. The original theory needs 117 tests in dimension d = 3, and it is complex and nearly impossible to demonstrate experimentally. Afterwards the theory is simplified by many researchers^2^,^3^,^4^,^5^,^6^,^7^. These simplified contextuality theories have been tested experimentally, for instance in photon^8^,^9^,^10^,^11^,^12^,^13^, neutron^14^,^15^, trapped ion^16^ and nuclear magnetic resonance^17^,^18^ systems. For the three-level system, Yu and Oh proposed a contextuality inequality, which needs only 13 variables and 24 pair correlations^19^. The scenario is state independent and only requires a single qutrit. Klyachko, Can, Binicioglu, and Shumovsky also found a state dependent contextuality inequality in the three-level system^20^. The three-level system is the simplest case to show quantum contextuality. The Klyachko-Can-Binicioglu-Shumovsky (KCBS) inequality is fundamentally important, because it is behind the violation of other noncontextualities^21^.

The state independent inequality (13 variables and 24 pair correlations as described in ref. 19) has been demonstrated by Zu *et al*. using the qutrit encoded in three paths of the single photons^22^. In a single trapped ion system, the inequality has also been tested by Zhang *et al*.^23^. Using the polarization and path of single photons, the demonstration of the inequality has been implemented by Huang *et al*.^24^. These three-level systems are indivisible. For the KCBS inequality as shown in ref. 20, several experiments have also been performed with the single photons, trapped ions and biphotons. Lapkiewicz *et al*. firstly proved the violation of the KCBS inequality with the single photon qutrit^25^. Subsequently, the experimental demonstrations of the KCBS inequality and the Wright inconsistency have been reported by Ahrens *et al*.^26^. The demonstration of violation of the KCBS inequality and the random-number production have been implemented by a single trapped ion^27^. Using nitrogen-vacancy centers, Kong *et al*. have tested the violation of the geometrical form of the KCBS inequality^28^. An experiment for the KCBS contextuality and nonlocality has also been implemented by Shaham *et al*.^29^. In addition, the no-disturbance monogamy relation about the KCBS contextuality and the nonlocality has also been discussed^30^,^31^. The contextuality is important for applications^32^ and theoretical investigations, but it is difficult to implement experimentally. Thus, it is necessary to develop new simplified methods.

On the other hand, recent investigation has shown that the quantum bound is not exclusive to quantum theory^33^. It has been demonstrated that the quantum bound exists similarly in classical microwaves. The local and nonlocal correlations in the classical optical beams, which violate the Bell inequality, have been demonstrated in a series of works^34^,^35^,^36^,^37^,^38^,^39^,^40^,^41^. However, the KCBS contextuality has not been studied in classical wave systems so far.

In this paper we study the KCBS contextuality in classical light systems. The polarization and path of the classical light beams are used to build the classical trit, which is called the “cetrit” like the cebit in ref. 42. Based on the bases of the cetrit, the input state is constructed, and then the observables are tested by projective measurement. Here the typical KCBS inequality and its geometrical form are studied, and the results are compared with the noncontextuality cases.

## Results and Discussion

### KCBS inequality and its expression in classical optical systems

According to ref. 20, if we consider the five observables 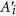 (*i* = 1, 2, …. 5), which have the values +1 or −1, the algebraic inequality for any choice of them can be expressed as


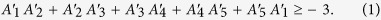


For the contextuality inequality, the observables 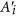 correspond to the five unit vectors, which are expressed with 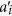. The eigenvalues of the operators 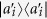 are 0 or 1, as well as 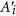 = 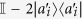 (where 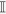 is a 3 × 3 identity matrix), thus the eigenvalues of 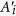 are +1 or −1. The unit vectors 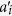 are presented at the five vertexes of pentagram, where 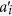 and 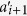 (*i* + 1 modulo 5) are orthogonal, and they are compatible. When 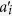 and 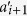 project to the pentagram plane, the angle between the projected vectors of 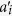 and 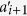 is 4π/5, and the angle between the direction vector and the symmetry axis is approximately 4π/15. It is easy to obtain:


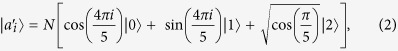


where 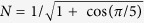 is a normalization constant. For the KCBS inequality, the input state is at the symmetry axis of the pentagram^20^. The input state |*φ*〉 projects to the five couples of observables 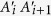, and the inequality has the maximum violation,


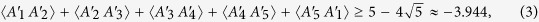


where 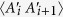 is used to denote the average of the measurement outcome of 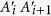. The KCBS inequality is state dependent, here the input state is taken as the specific input state |*φ*〉 = |2〉. The geometric form of the KCBS inequality or Wright’s inequality is expressed as^20^


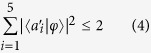


Under the Kochen-Specker rules, the value at every direction vector is predetermined to 1 or 0, but the maximum is only one for the two orthogonal directions. Thus, the maximum is two for the five directions of pentagram. In quantum mechanics we select the same input state like that in the KCBS inequality, and 
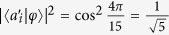
 (the angle between 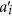 and |*φ*〉 is approximately 4π/15) can be obtained. Thus, we have


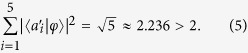


The above inequalities (Equations (3) and (5)) have been demonstrated experimentally using quantum states^25^,^26^,^27^,^28^,^29^.

**Figure 1:**
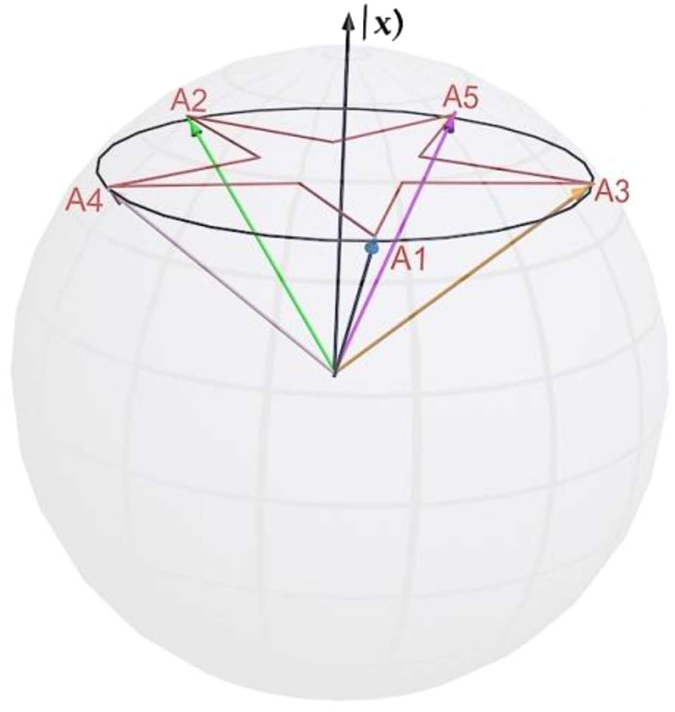
Representation of the measurement for the KCBS inequality in three-dimensional space. *A*_1_, *A*_2_, *A*_3_, *A*_4_, *A*_5_ are observables at five directions, and the direction vectors are pairwise orthogonal. |*χ*) is used to express the input state in the classical light systems.

Now we use classical optical fields to express the input state and the direction unit vectors for the KCBS inequality and its geometric form as showed in Fig. 1. The input state is written as 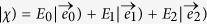, where *E*_0_, *E*_1_ and *E*_2_ represent amplitudes of the classical optical fields, and 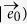, 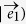 and 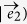 are the cetrit’s bases corresponding to quantum bases |0〉, |1〉 and |2〉 in three-dimensional space^43^. Here a slightly modified version of the familiar bra-ket notation of quantum mechanics is taken to express these vectors in the classical optical fields. Meanwhile the direction unit vector can be denoted as 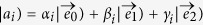, where 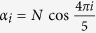, 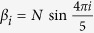 and 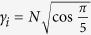 are the coefficients corresponding to those in Eq. (2).

In the following we calculate the correlations *A*_*i*_*A*_*i*+1_ of classical optical fields. In order to describe the KCBS inequality, 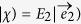 is taken as the input state, which is at the symmetry axis of the pentagram as shown in Fig. 1. 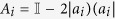 is used to express the observable in the classical optical systems, where |*a*_*i*_) is the direction unit vector. For the input state |χ), from the correlation 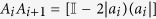 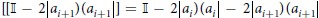, we can obtain the average value (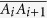) of *A*_*i*_*A*_*i*+1_ as 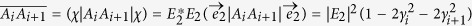

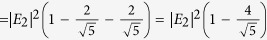
. The KCBS inequality requires the specific input state, thus the amplitude *E*_2_ need to be normalized. When it is normalized, the value 
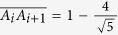
, therefore


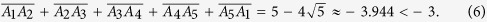


Equation (6) corresponds to Eq. (3), which is the corresponding form of the KCBS inequality in the classical optical systems. For the geometric form of the KCBS inequality (or called Wright’s inequality), the input state is 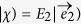, similarly we can obtain the equation 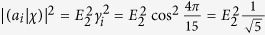. The amplitude of the optical field needs to be normalized, 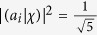, thus


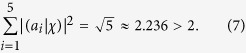


The geometric form of the KCBS inequality is also violated in the classical optical systems. In the following, we test experimentally the KCBS inequality and its geometrical form in the classical light systems.

### Experimental demonstration of the KCBS inequality in classical light systems

**Figure 2:**
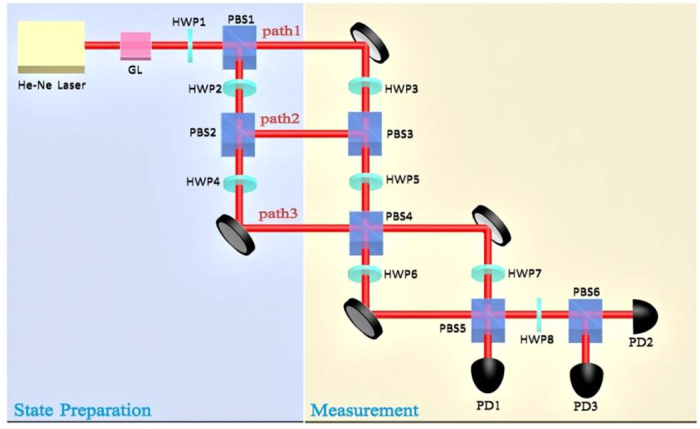
The experimental setup for the test of the KCBS inequality. The laser pulses from the He-Ne laser transmit through the GRIN lens (GL), and we obtain the horizontal polarization optical beam. Then the beam is modulated by HWPs and PBSs to achieve our construction. At last the light intensities are detected by the three photoelectric detectors (PD1, PD2 and PD3) at the three output ports. The HWP3, 4, and 6 are used for path-length compensation. HWP, half-wave plate; PBS, polarizing beam splitter; PD, photoelectric detector.

The test of the KCBS inequality needs a three-level system. Using classical optical beams, we can construct the corresponding system. The scheme is shown in Fig. 2. It consists of two parts: state preparation and measurement. In the state preparation stage, the laser beam from He-Ne laser transmits through Glan prism and then the laser beam possessing the certain polarization appears. Here the center wavelength of the laser beam is 633 nm. The beam is split through polarizing beam splitters (PBSs) and is modulated by half-wave plates (HWPs) to perform the construction of classical trit (cetrit). The different polarizations in three paths of classical optical fields are used to carry out the process as showed in Fig. 2. The horizontal polarization for the first path, the vertical polarization for the second path, and the horizontal polarization for the third path are encoded to 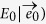, 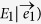 and 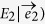, respectively, that is


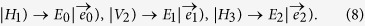


where 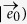, 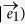 and 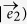 are the basis vectors, *E*_0_, *E*_1_ and *E*_2_ represent the amplitudes of classical optical fields for the different paths. With tuning the HWP1 and HWP2, the different amplitudes of classical optical fields in three paths can be obtained, thus the desired input state can be represented with these bases, namely, 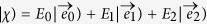. In our experiment, the HWP1 and HWP2 all are set to 45°, and the specific input state 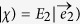 for the KCBS inequality is obtained.

In the KCBS experiment, these observables correspond to their respective operators. After the input state is projected onto the eigenstates of each operator, the measurement for the observable can be implemented. In the three-level system, the operator owns three different eigenstates and the corresponding eigenvalues. Here the input state is projected onto the eigenstate and then the probability of the eigenvalue is obtained. When the eigenvalue is multiplied by its probability, we can obtain the observable by summing the product. Nevertheless, the probability can be obtained by detecting the light intensity in our classical optical experiments (see Methods section for details).

For the KCBS inequality, we need to test the correlation 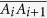, and the observables *A*_*i*_ and *A*_*i*+1_ need to be tested simultaneously. Here the operator 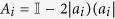, and its eigenvalues are +1, +1 and −1, which correspond to the values of 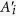 in Eq.(1). The eigenvalues +1 and −1 for the operator 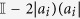 correspond to the eigenvalues 0 and 1 for |*a*_*i*_)(*a*_*i*_|, and their eigenstates are also one-to-one correspondence. We adopt the scheme of joint measurement to test the contextuality inequality^22^. In order to measure the correlation *A*_*i*_*A*_*i*+1_, we need to know their respective eigenstates corresponding to eigenvalues +1, +1 and −1. As *A*_*i*_ and *A*_*i*+1_ are orthogonal, the eigenstate of *A*_*i*_ corresponding to the eigenvalue −1 is just the eigenstate of *A*_*i*+1_ corresponding to the eigenvalue +1. In contrast, for the eigenstate corresponding to *A*_*i*+1_ = −1, it is just the eigenstate corresponding to *A*_*i*_ = +1. When the input state is projected onto the respective eigenstates, we can obtain the joint probabilities *P(A*_*i*_ = −1, *A*_*i+*1_ = +1) and *P(A*_*i*_ = +1, *A*_*i+*1_ = −1. At the same time, there is the third eigenstate that the eigenvalues all are +1 for *A*_*i*_ and *A*_*i*+1_, and the probability is *P(A*_*i*_ = +1, *A*_*i+*1_ = +1. However, the eigenstate is nonexistent when the corresponding eigenvalues for both *A*_*i*_ and *A*_*i*+1_ are taken as −1, there is no the joint probability in such a case. With these probabilities, we can obtain 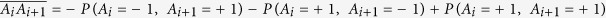. Then, the contextuality can be calculated from Eq. (6).

The above measurement process can be completed by the experimental setup of measurement part in Fig. 2. For the measurement of the observable *A*_*i*_*A*_*i*+1_, because *A*_*i*_ and *A*_*i*+1_ possess a joint probability, they are required to measure simultaneously in the experiment. Due to the complexity of expressions for *a*_*i*_, it is difficult to establish the eigenstates of *A*_*i*_ and *A*_*i*+1_ simultaneously. The experiment setting needs to be designed skillfully and the observable *A*_*i*_*A*_*i*+1_ needs to be measured carefully. In order to obtain the eigenstates of *A*_*i*_ and *A*_*i*+1_ simultaneously, some designs are done and several interferometers are cascaded to achieve the above purpose. Then the cetrit bases can map to these eigenstates at the three output ports as showed in Fig. 2. Using the HWPs (5, 7 and 8) and PBSs (4, 5 and 6), the polarization and path are combined to accomplish the construction. The expressions of the state vectors at the three output ports are showed in Eq. (11) (see Methods section).

With suitably setting up the angles of HWP5, HWP7 and HWP8, the eigenstates of these observables can be obtained at three output ports. They meet the requirements of joint measurement. Taking *A*_1_ and *A*_2_ for examples, the setting angles of HWP5, HWP7 and HWP8 are 117°, 24°, and 32°, respectively. Hence the state vectors at output ports 1, 2 and 3 are 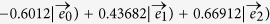, 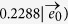

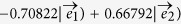
, 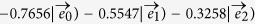, respectively. The output states at the port 1 describe the eigenstates with eigenvalues *A*_1_ = −1and *A*_2_ = +1. The output states at the port 2 correspond to the eigenstates with *A*_1_ = +1 and *A*_2_ = −1. But the output states at the port 3 are the eigenstates with *A*_1_ = +1 and *A*_2_ = +1. The state vectors of output ports 1 and 2 correspond to the expressions in Eq. (9) (see Methods section), and they are the interrelated eigenstates of *A*_1_ and *A*_2_. Because of the imperfect setting angles of HWPs, the expressions have a little deviation. With the input state being projected onto these eigenstates, the joint probabilities are obtained when we measure the light intensities at the three output ports. The light intensities are detected by three photodetectors (PD1, PD2 and PD3). The light intensity of each output port is normalized (divided by the total light intensities), and the probability of eigenvalue is obtained. The measurement probabilities for the output ports 1, 2 and 3 correspond to *P(A*_1_ = −1, *A*_2_ = +1), *P(A*_1_ = +1, *A*_2_ = −1) and *P(A*_1_ = +1, *A*_2_ = +1), respectively, thus the observable *A*_1_*A*_2_ can be obtained.

The measurement methods for all five pairs of observables are the same. We tune the angles of the HWPs and obtain the desired eigenstates of the pairs of observables. The setting angles of the HWPs for the measurements of the different correlation pairs are given in Methods section. Then the input state is projected onto these eigenstates and the light intensities of the output ports are measured for every setting. The light intensity of each output port is normalized, therefore the joint probabilities of *A*_*i*_ and *A*_*i*+1_ are acquired, *P*_PD1_ = *I*_1_/(*I*_1_ + *I*_2_ + *I*_3_), *P*_PD2_ = *I*_2_/(*I*_1_ + *I*_2_ + *I*_3_) and *P*_PD3_ = *I*_3_/(*I*_1_ + *I*_2_ + *I*_3_). And then using the relation: 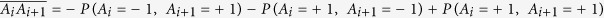, the five pairs of observables can be calculated. The probabilities and measurement values of 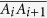 are given in the following Table 1.

With summing the five sets of results, we can calculate the contextuality for Eq. (6). The result is −3.4196 ± 0.0057 and less than the minimum value −3. Due to inaccuracy of the experiment measurements, the experiment results have some deviations from the theoretical values. However, they still show strong violation of the noncontextuality inequality. This means that the KCBS contextuality can be realized in the classical optical systems similar to the cases in the single photon systems.

### Experimental demonstration for the geometric form of the KCBS inequality in classical light systems

**Figure 3:**
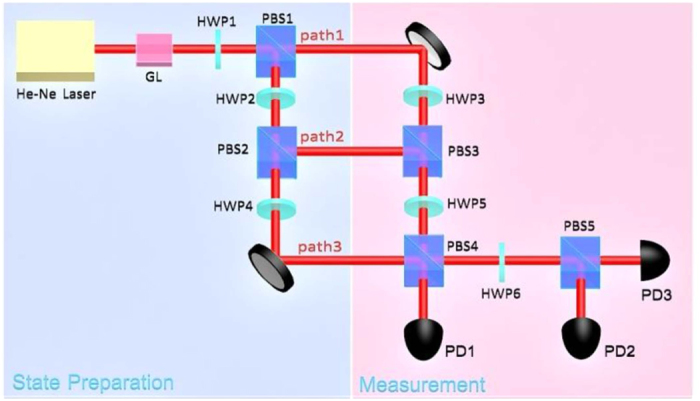
Experimental setup for the test of the geometric form of the KCBS inequality. The establishment of the input state and the eigenstates, and the detection method are similar to the test of the KCBS inequality. The HWP3 and HWP4 are used for the path-length compensation similarly.

For the geometric form of the KCBS inequality, the input state needs to be projected onto the eigenstates of the operator |*a*_*i*_)(*a*_*i*_|. The operator owns eigenvalues 0, 0, +1 and the corresponding eigenstates. When the input state is projected onto the relevant eigenstates, the probability of the eigenvalue is obtained. Each eigenvalue is multiplied by its respective probability. Then with summing the three computation results, we got these observables. In such a case, the experimental setting shown in Fig. 2 can be simplified to Fig. 3 to measure the geometric form of the KCBS inequality.

In the state preparation stage as showed in Fig. 3, the different polarizations of the classical light fields in three paths indicate three-dimensional basis vectors. Just like the case in the Fig. 2, these bases construct the input state 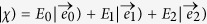. Likewise, the angles of the HWP1 and HWP2 all are set to 45° to obtain the particular input state 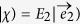 for the geometric form of the KCBS inequality.

Similarly we need to construct these eigenstates of the operator |*a*_*i*_)(*a*_*i*_| and implement the projective measurements in the measurement stage. As showed in Fig. 3, with utilizing the appropriate settings for HWP5, HWP6 and the PBSs, the three eigenstates of |*a*_*i*_)(*a*_*i*_| can be constructed using the classical optical fields at the three output ports. The state vectors at the three output ports are expressed in Methods section. The output ports 1, 2 and 3 correspond to the eigenstates with eigenvalues 0, 0 and +1, respectively. Take |*a*_1_)(*a*_1_| as an example, the setting angles of HWP5 and HWP6 are 117° and 24°, respectively. We assume that the amplitudes of the three input bases all are unit amplitude, the light fields can expressed as 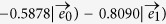, 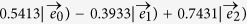, and 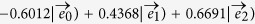 at the three output ports 1, 2 and 3, respectively. They correspond to the three eigenstates with eigenvalues 0, 0 and + 1 of the operator |*a*_1_)(*a*_1_|. In order to obtain the probabilities corresponding to eigenvalues 0, 0 and +1, we measure the light intensities at the respective output ports. After the normalization, we obtain the probabilities of eigenvalues, namely *P*_PD1_ = *I*_1_/(*I*_1_ + *I*_2_ + *I*_3_), *P*_PD2_ = *I*_2_/(*I*_1_ + *I*_2_ + *I*_3_) and *P*_PD3_ = *I*_3_/(*I*_1_ + *I*_2_ + *I*_3_). With the probabilities we can calculate the value 
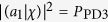
 and further obtain 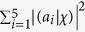. Of course, the light intensity may also be regarded as the modular square of the input state |χ) being projected directly onto the direction vector *a*_*i*_, which corresponds to the expression in Eq. (7). For the other terms, the methods are the same. The Eq. (7) can be calculated using these measurement results and then we can detect the violation of the geometric form of the KCBS inequality. The experimental results are shown in Table 2.

The upper bound of the inequality for Eq. (4) is 2 and the maximum theoretical prediction is 2.236. Our experiment result is 2.1880 ± 0.0064, which show obvious violation of the inequality. This means that the violation of the geometric form of the KCBS inequality can also be observed in classical light systems. The above experimental results of the KCBS inequality and its geometrical form have always some deviations with the theoretical maximum violation. This is because there are some imprecise measurements and imperfect operations on optical elements in the experiments. For example, the PBS cannot transmit the horizontal polarization light and reflect the vertical polarization light absolutely. The mixed polarization lights always enter into the light path because of the inaccurate PBS. Although there are some imprecise measurements, the presented experiments still yield strong violation of the KCBS inequality and its geometrical form.

## Conclusions

We have performed the experimental test for the KCBS inequality and its geometry form (or Wright’s inequality) in the classical light systems. Using the polarization and path, the cetrit has been constructed, the projection measurement has been implemented, the clear violations of the KCBS inequality and its geometrical form have been observed. This indicates that the contextuality inequality, which is commonly used in test of the conflict between quantum theory and noncontextual realism, can be used in the classical optical coherence to describe correlation characteristics of the classical fields. In addition, recent investigations have shown that there is a remarkable equivalence between the onset of contextuality and the possibility of universal quantum computation^32^. Thus, our results also imply that the strong quantum computing power is likely to be simulated in the classical light systems.

## Methods

### The probability of eigenvalue is tested by measuring light intensity in classical optics systems

In the classical optical systems, the direction unit vector can be expressed as 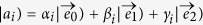, and in the general case the input state is 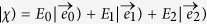. In Eq. (2) the expression of the state vector is clear for the geometric comprehension of pentagram direction unit vector, but it is not direct for the algebra calculation. Thus, we transform it into the decimal forms:


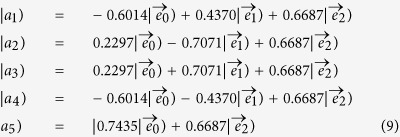


In the measurement stage, the input state needs to be projected onto the different eigenstates of the operator *A*_*i*_, and then we can calculate the observables. Here the eigenstates are also expressed with the classical optical fields, where |*a*_*i*_) is exactly the eigenstate corresponding to the eigenvalue −1 for the operator *A*_*i*_ (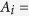

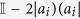
). The probability for the eigenvalue −1 is 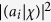 when the input state |*χ*) is projected onto the eigenstate |*a*_*i*_). The probability |(*a*_*i*_|χ)|^2^ is the modular square of the amplitude of the optical field in the experiment, and the modular square of the amplitude is the light intensity *I*


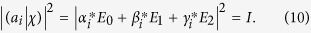


Thus the probability of the eigenvalue can be expressed with the light intensity and we can detect the light intensity to obtain the probability of the eigenvalue in our experiment. The probabilities of two other eigenvalues can be measured similarly.

### The expressions of state vectors at the three output ports and the setting angles of HWPs for the two experiments

For the KCBS inequality, we need to construct the interrelated eigenstates that satisfy the joint measurement scheme at the three output ports. The transformation matrix of the HWP is 
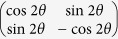
, where *θ* is the rotation angle of the HWP. We assume that all amplitudes for the three input bases are unit amplitudes, and at the three output ports the output expressions may be written as


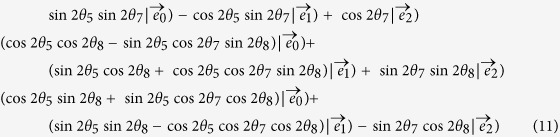


In the Table 3, we display the rotation angles of HWPs for the measurements of five pairs of observables for the KCBS inequality. For the geometric form of the KCBS inequality, we need to construct the eigenstates of the operator |*a*_*i*_)(*a*_*i*_| with the combination of HWPs and PBSs. As showed in Fig. 3, at the three output ports 1, 2 and 3, the state vectors can be expressed as 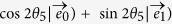, 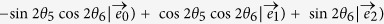 and 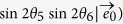

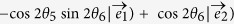
, respectively. With suitably choosing the angles of HWP5 and HWP6, the desired eigenstates are obtained at the three output ports. In the following Table 4, it is the setting angles of HWP5 and HWP6 for the five measurements.

## Additional Information

**How to cite this article**: Li, T. *et al*. Experimental contextuality in classical light. *Sci. Rep.* **7**, 44467; doi: 10.1038/srep44467 (2017).

**Publisher's note:** Springer Nature remains neutral with regard to jurisdictional claims in published maps and institutional affiliations.

## Figures and Tables

**Table 1:** The experimental probabilities and calculated results for the KCBS inequality.

Terms	PD1		PD2		PD3		Calculated result
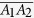	*P*( 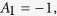 *A*_2_ = +1)	0.4308 ± 0.0046	*P(A*_1_ = +1, *A*_2_ = −1)	0.4204 ± 0.0049	*P(A*_1_ = +1, *A*_2_ = +1)	0.1488 ± 0.0012	−0.7024 ± 0.0024
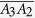	*P(A*_3_ = −1,*A*_2_ = +1)	0.4313 ± 0.0039	*P(A*_3_ = +1, *A*_2_ = −1)	0.3974 ± 0.0053	*P(A*_3_ = +1, *A*_2_ = +1)	0.1713 ± 0.0023	−0.6574 ± 0.0046
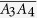	*P(A*_3_ = −1,*A*_4_ = +1)	0.4313 ± 0.0026	*P(A*_3_ = +1, *A*_4_ = −1)	0.4154 ± 0.0027	*P(A*_3_ = +1, *A*_4_ = +1)	0.1533 ± 0.0036	−0.6935 ± 0.0071
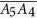	*P(A*_5_ = −1,*A*_4_ = +1)	0.4297 ± 0.0029	*P(A*_5_ = +1, *A*_4_ = −1)	0.4011 ± 0.0068	*P(A*_5_ = +1, *A*_4_ = +1)	0.1691 ± 0.0042	−0.6618 ± 0.0084
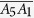	*P(A*_5_ = −1,*A*_1_ = +1)	0.4313 ± 0.0045	*P(A*_5_ = +1, *A*_1_ = −1)	0.4210 ± 0.0035	*P(A*_5_ = +1, *A*_1_ = +1)	0.1477 ± 0.0012	−0.7045 ± 0.0024
							−3.4196 ± 0.0057

**Table 2:** The experimental results and the theoretical predictions of the observables for the geometric form of the KCBS inequality.

Terms	PD1 *P(a*_*i*_ = 0)	PD2 *P(a*_*i*_ = 0)	PD3 *P(a*_*i*_ = 1)	Theoretical value
*a*_1_	0.0381 ± 0.0004	0.5214 ± 0.0019	0.4405 ± 0.0021	0.4472
*a*_2_	0.0402 ± 0.0003	0.5232 ± 0.0020	0.4366 ± 0.0019	0.4472
*a*_3_	0.0388 ± 0.0003	0.5239 ± 0.0014	0.4373 ± 0.0014	0.4472
*a*_4_	0.0401 ± 0.0007	0.5214 ± 0.0031	0.4386 ± 0.0034	0.4472
*a*_5_	0.0395 ± 0.0003	0.5255 ± 0.0030	0.4350 ± 0.0029	0.4472
			2.1880 ± 0.0064	2.236

Because the eigenvalues for the output port 1 and 2 all are 0, the result of the output port 3 is the value of the observable.

**Table 3:** The setting angles of the HWPs for the test of the KCBS inequality.

	HWP5	HWP7	HWP8
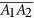	117°	24°	32°
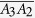	81°	24°	58°
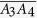	81°	24°	122°
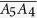	45°	24°	148°
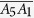	45°	24°	32°

**Table 4:** The setting angles of HWP5 and HWP6 for the test of the geometric form of the KCBS inequality.

	*a*_1_	*a*_2_	*a*_3_	*a*_4_	*a*_5_
HWP5	117°	9°	81°	−27°	45°
HWP6	24°	24°	24°	24°	24°
